# Lend Me a Hand: A Value-Based Care Case Study on Pan Plexopathy of Unknown Origin

**DOI:** 10.7759/cureus.20354

**Published:** 2021-12-12

**Authors:** Victoria J Siu, Thomas Varkey, Umer N Khan, Jack B Ding, Saurin Gandhi

**Affiliations:** 1 Internal Medicine, Dell Medical School, Austin, USA; 2 Colangelo College of Business, Grand Canyon University, Phoenix, USA; 3 Neurology, Dell Seton Medical Center, Austin, USA; 4 Internal Medicine, University of Adelaide, Adelaide, AUS; 5 Internal Medicine, Royal Adelaide Hospital, Adelaide, AUS

**Keywords:** pocus (point of care ultrasound), brachial plexus injury, imaging modalities, adult neurology, value based care

## Abstract

This paper discusses an interesting case of pan plexopathy and the difficulties associated with the diagnostic processes based on patient-specific circumstances. It walks through the major differential of the etiology of the patient’s particular presenting symptoms and the associated diagnostic and therapeutic process by which this particular patient was treated. In the discussion, the relevant anatomy of the brachial plexus and the surrounding structures in both the cervical and the axillary regions is discussed and key clinical pearls that became apparent throughout the diagnostic workup that was significant for a hematoma and therapeutic process aimed at providing symptomatic relief until recovery to baseline. This case study discusses the benefits, drawbacks, and financial costs of utilizing the major different imaging modalities such as CT, MRI, or Point of Care Ultrasound (POCUS). Finally, this study provides a new diagnostic algorithm for the selection of the imaging modality based on the major principles of value-based care as detailed by both the Radiological Society of North America and the European Society of Radiology.

## Introduction

The brachial plexus provides sensation and motor control for the upper extremities [1]. Loss of the entire brachial plexus is catastrophic for a patient’s quality of life and hampers activities of daily living [2]. This case study discusses the clinical presentation of a 50-year-old male who presented to the level 1 trauma facility with right arm paresthesias and weakness two weeks after being discharged from the intensive care unit (ICU) at a different facility where he was being treated for pneumonia and required an inferior jugular central line during his stay. We aim to highlight the diagnostic workup, the treatment of the patient’s particular condition, and clinical pearls for evaluating patients with regards to the diagnostic imaging and the expenses associated with those imaging studies.

## Case presentation

The patient is a 50-year-old male who was transferred from his long-term rehabilitation facility to a level 1 trauma facility with right arm paresthesias and weakness. His past medical history was pertinent for atrial fibrillation, hypertension, and a recent pneumonia episode which had required a stay in the intensive care unit roughly two weeks before admission to the level 1 trauma facility.

The internal medicine team consulted the neurology team to further evaluate the complaints of right arm numbness and weakness. The internal medicine team stated that they were fearful about the patient’s neurologic functional status. History was derived from the patient's wife and daughter because the patient had a tracheostomy tube. 

The patient was hospitalized two weeks ago at a nearby hospital where he was being treated for pneumonia. During his stay, a central line was placed through the right internal jugular vein and was removed prior to discharge. The patient first complained of numbness and weakness from the level of the lateral epicondyle to the distal end of the third digit a few days after he was discharged from the ICU at a different facility. The symptoms of weakness and pain progressively worsened over the next few days. This led to a complete loss of sensory and motor function in the entire right upper extremity from the level of the shoulder to the distal end of the third digit. Although the patient reported complete loss of sensation as stated above, the patient did endorse paresthesias and burning pain in his right upper extremity. 

On general inspection, the patient was a well-groomed individual measuring 73 inches (6 foot 1 inch) and 212 kg. Power in the left upper limb was 5/5, with normal sensation to two-point pinprick and light touch. He had normal 2+ reflexes on the left upper extremity and in the bilateral lower extremities. His right biceps, triceps, and brachioradialis reflex were all negative. Babinski sign and the Hoffman reflex were negative bilaterally. On the right side, the patient had 0/5 strength throughout. He was unable to sense soft or dull touch in the affected regions. 

Differential diagnosis

Based on the history and physical examination, the working diagnosis was determined to be brachial pan plexopathy. This was due to the loss of both motor and sensory at the level of the upper arm all the way down the right upper extremity to the level of the fingertips and loss of reflexes on the affected side. These physical exam findings are indicative of a lower motor neuron deficit leading the team to give the working diagnosis of pan-plexopathy or a loss of function for the brachial plexus. The differential for the etiology of this pan-plexopathy included viral infection, mechanical injury secondary to compression or insertion of the inferior jugular intravenous catheter, inflammation of the brachial plexus or other structures secondary to the patient’s clinical condition, and/or an amalgamation of multiple or all of the differential as listed. Based on the differential, it was determined that imaging of the affected arm was the next most appropriate step.

Story continues

Due to loss of the entire extremity’s function, neuroradiology recommended further assessment with computerized tomography (CT) head and cervical spine. The patient’s weight exceeded the safety limitations for performing standard magnetic resonance imaging (MRI). Furthermore, the patient’s body habitus and weight exceeded safety limitations for utilizing standard-sized CT machines. The next recommended line of imaging was open MRI, which was unavailable at this facility. Neuroradiology suggested transferring the patient to the closest available open MRI machine at the neighboring city zoo 100 miles away. However, expenses and logistics of transportation, stigma of utilizing zoo facilities, and the patient’s state created an expensive and unrealistic option for altering management.

As an alternative to transporting the patient to the neighboring city zoo 100 miles away, a neurology resident suggested that the patient undergo point of care ultrasound of the neck and axillary region. However, the neuroradiology team stated any discoverable findings through this modality would not alter treatment. The neuroradiologist on service further stated that the utilization of an electromyographic (EMG) machine would not provide any pertinent details for at least two weeks after the initial insult. The discussion on ideal selection of local imaging services or transportation to a nearby city continued for three days, and was further complicated by the patient’s lack of access to funding for transportation without significant evidence of altering treatment outcome.

End diagnosis

Following three days of discussion, the neurology team proposed a point of care ultrasound (POCUS) as a first-line imaging choice. The rationale was POCUS was readily accessible at the facility, served as an excellent screening tool, and may provide pertinent information which could affect management of the patient. While neuroradiology disagreed with the plan, the POCUS demonstrated a deep pocket of fluid. This pocket of fluid was later demonstrated to be a hematoma secondary to the removal of an inferior jugular central line which expanded over the course of two weeks. This finding coincided with the patient’s onset of symptoms within a few days of removal of the central line upon discharge from the intensive care unit. During the work-up of his symptoms, the patient reported some return of sensation and motor function at the level of the proximal and distal interphalangeal joints. Which confirmed the diagnosis as repeat POCUS imaging demonstrated a decrease in the size 

Treatment

Following completion of the work-up, the primary and neurology teams agreed conservative monitoring was key in the patient’s care, given the self-resolving nature of the symptoms. He was continued on 400 mg Gabapentin dosed three times daily orally to mitigate neuropathic pain during recovery. He was encouraged to continue elevating his arm to prevent the pooling of venous blood flow in the extremity. The care team encouraged him to work with physical and occupational therapy for several hours a day to regain arm strength. When the patient was discharged, he was given orders for an outpatient neurology and EMG study follow-up to ensure full recovery of his neurological function in the right upper extremity.

## Discussion

Communication with the patient was complicated due to the impairment of his voice and writing hand. As a result, including family members in the discussion enabled efficient and targeted history taking, which was critical. Involving the patient’s family and friends in discussions may be important for providing an alternative view on the matter, and in this case, proved crucial for providing the primary view of the patient’s disease severity and how different treatment strategies may affect the patient’s overall quality of life [3]. This can be applied to situations when the language spoken by the provider is not the first language of the patient according to a chart review study in 2019 [4] or if the patient demonstrates signs of cognitive impairment from dementia or other processes as suggested by studies in 2016 [5] and 2018 [6]. Often in circumstances where the patient is unable to provide history, collecting collateral history becomes the only method to collect information [7].

**Figure 1:**
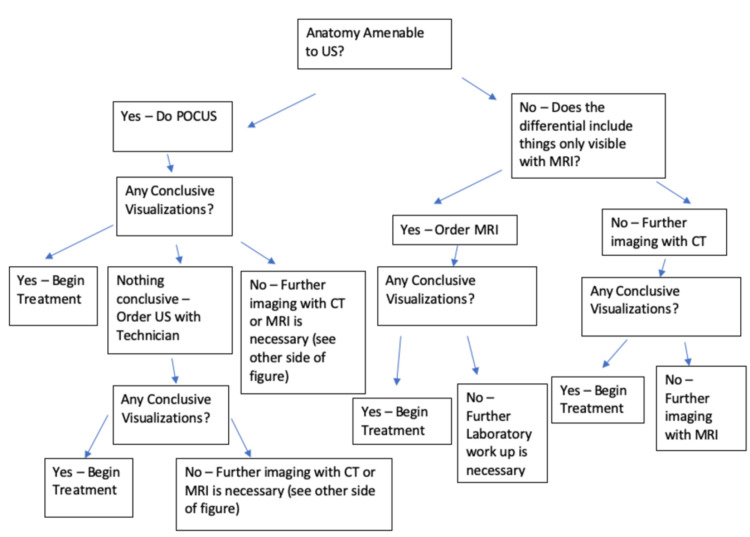
Value-based care imaging diagnostic tree. US: ultrasound; CT: computerized tomography; MRI: magnetic resonance imaging. This algorithm was based on recommendations of general principles from both the Radiological Society of North America and the European Society of Radiology [18,19].

Value-based care focuses on only ordering tests to rule in or out items on the differential generated secondary to the information gathered during the patient’s subjective history and objective physical examination [8]. While often misunderstood as not being patient-centered, value-based care asks the question, “Is there another way to get the same information about the patient’s clinical condition? If so, which test provides the most accurate and precise information?” This focus on only doing what is necessary and helpful defines patient-centered care [9]. In the case study, a CT required the patient to be transported by ambulance over 100 miles, an unaffordable cost for the patient, to receive the same end diagnosis that was easily obtained through POCUS. Included is a table which compares the utilization, drawbacks, and costs of different imaging studies for the patient (see Table 1). Across the literature, the highest recommendation of the authors is for all diagnostic choices to be driven by the history and physical examination first. However, the old adage, “start low and go slow” can and does often apply. Therefore, a small flow chart is included for review (see Figure 1).

**Table 1:** Benefits, drawbacks, and cost of different imaging studies. This table provides information of the uses of the different imaging studies, drawbacks, and the overall cost to the patient, *cost is based on national average, **billing information on POCUS not widely available with large variations depending on region and hospital setting.

Study	Benefit	Drawback	Cost to patient*
Point of care ultrasound (POCUS)	High temporal resolution, fast, portable, readily available [11,12], no radiation exposure [13]	Operator dependent, positioning and body habitus limits image acquisition, lower spatial resolution than MRI and CT [11-13]	$0 - $5** [14]
Ultrasound with technician	Same as the POCUS, but with increased technical skills from the operator of the imaging study [11, 13]	Slower than POCUS, positioning and body habitus limits image acquisition, lower spatial resolution than MRI and CT [11,13]	$110 - $370 [15]
Computerized tomography (CT)	More spatially detailed than US, next highest level of anatomic visualization after MRI [13]	Slower than US due to time-consuming workflow of ordering and acquiring imaging study, requires radiologist interpretation, moderate level of radiation exposure (1-10 mSv) [11,13]	$300 – $3,800 [16]
Magnetic resonance imaging (MRI)	Superior soft-tissue contrast, good spatial resolution, no radiation exposure [13]	Slower than CT due to lengthy workflow of ordering and acquiring imaging study, requires radiologist interpretation, contraindicated by certain implantable devices [11,13]	$400 – $5,700 [17]

The average American patient cannot afford an unexpected $400 bill. According to a report from the Federal Reserve Bank of Minneapolis released in June of 2021, nearly 50% of American families would have great difficulty paying for a $400 emergency expense and 19% would be unable to afford the cost whatsoever [10]. This becomes pertinent as a single hospital stay far outpaces even a $400 cost.

Review of pertinent anatomy

The brachial plexus is a network of nerves supplying motor and sensory innervation to the upper limb. It begins with the roots of the C5-T1 nerves in the lateral cervical region [1]. C5 and C6 roots merge into the superior trunk, C7 roots form the middle trunk, C8 and T1 roots merge into the inferior trunk [1]. The trunks each split into anterior and posterior divisions [1]. Anterior divisions of the superior trunk and middle trunk form the lateral cord [1]. Posterior divisions from the superior, middle, inferior trunks form the posterior cord [1]. Anterior division of the inferior trunk forms the medial cord [1]. Parts of the lateral and medial cord merge to form the median nerve while the rest of the lateral cord continues as the musculocutaneous nerve and the rest of the medial cord continues as the ulnar nerve [1]. The posterior cord gives rise to the axillary and radial nerves [1].

Roots and trunks of the brachial plexus are sandwiched between the anterior and middle scalene muscles in the posterior triangle of the neck [1]. The posterior triangle of the neck is outlined by the posterior border of the sternocleidomastoid forming the anterior boundary, the anterior border of the trapezius muscle forming the posterior boundary, and the middle one-third of the clavicle forming the inferior boundary [1]. The internal jugular vein sits above the anterior scalene muscles and acts as a continuation of the sigmoid vein, collecting blood from the brain and superior portion of the face which then drains into the right brachiocephalic vein and ultimately the right atrium [1].

The brachial plexus is susceptible to injury due to its long superficial course from the neck to the arm [2]. Upper plexus lesions typically involve C5-C6 nerve roots, affecting deltoid and biceps muscles as well as sensory changes extending below the elbow to the hand [1]. Lower plexus lesions typically involve C8-T1 nerve roots, affecting median and ulnar muscle innervations, hand weakness and sensory changes in most of the palmar hand and ulnar aspect of the dorsal hand [1]. The patient's history and physical exam findings were suggestive of pan plexopathy, including signs of upper and lower plexus lesions. He likely developed a substantially large right internal jugular vein hematoma which compressed and displaced the anterior scalene muscles. In turn, the roots and trunks of the right brachial plexus were compressed against the anterior and middle scalene muscles, contributing to his symptoms of pan plexopathy.

## Conclusions

The actions taken by the team ensured that the patient was able to have resolution of his symptoms and recovery of the use of his upper extremity without increasing the cost of his hospitalization. Through utilizing the principles of value-based care, astute clinicians can ensure that patients are treated appropriately with regard to their clinical condition and financial situation. Only in keeping the patient at the center of the care provided and ensuring that their biological, psychological, social, and financial needs are met can a therapeutic relationship properly ensure healing for the patients under the care of the healthcare team. It is the hope of the authorial team that the table and diagnostic flow chart will provide support to clinicians wishing to engage within the space of value-based care.
